# Succession of oral microbiota community as a tool to estimate postmortem interval

**DOI:** 10.1038/s41598-019-49338-z

**Published:** 2019-09-10

**Authors:** Kaikai Dong, Ye Xin, Fangqi Cao, Zhiwei Huang, Jing Sun, Min Peng, Wenbin Liu, Ping Shi

**Affiliations:** 1 State Key Laboratory of Bioreactor Engineering, East China University of Science and Technology, Shanghai, 200237 China; 2 Shanghai Key Laboratory of Crime Scene Evidence, Shanghai Research Institute of Criminal Science and Technology, Zhongshan North No 1 Road, Shanghai, 200083 China; 3 Key Lab of Science & Technology of Eco-textile, Ministry of Education, College of Chemistry, Chemical Engineering and Biotechnology, Donghua University, 2999 Renmin Road, Shanghai, 201620 China; 4 Qinghai Key Laboratory of Qinghai-Tibet Plateau Biological Resources, Northwest Institute of Plateau Biology, the Chinese Academy of Sciences, Xiguan Avenue 59, Xining, 11, Qinghai Province 810001 China

**Keywords:** Applied microbiology, Metagenomics, Microbial ecology

## Abstract

The establishment of postmortem interval is one of the most important aspects of forensic expertise. Microbes may provide a novel way to estimate the postmortem intervals in order to avoid many of these limitations. The oral cavity harbors one of the most diverse microbiomes that play a key role in the decomposition of corpses. In this study, the oral bacterial community showed obvious changes in relative abundance during the process of mice decomposition. Meanwhile, at different taxonomic levels, specific bacteria were found to be significantly correlated with the postmortem interval. Linear regression models between relative abundance and the postmortem interval were constructed. Among these species, Gamma-proteobacteria and *Proteus* were the best ones that can be used to infer the postmortem interval, especially late postmortem interval. Therefore, we suggest that succession of oral microbial community can be developed as a forensic tool for estimating the postmortem interval.

## Introduction

Death is defined as the cessation of physiological processes that maintain cell integrity and function. Almost after death, the body begins to undergo an irreversible, ineluctable and progressive sequence of physical and chemical changes^1^. Understanding the expected autopsy changes is critical to the correct interpretation of the gross and micropathology of autopsy. In addition, the postmortem interval (PMI) estimate, which is the time after death, depends on the understanding of these postmortem processes to a large extent. It is critical to accurately estimate the PMI in forensic and law enforcement because it contributes to the identification of victims and suspects, the ascertainment or elimination of suspect witnesses, the notification of death certificates, and the distribution of assets listed in wills^2^. However, the PMI inference method is difficult to be established because PMI is susceptible to many external and environmental factors, such as temperature, humidity, oxygen tension, insects and scavenger activity^3^.

Traditional methods of estimating PMI include the following: (1) Gross Changes. During the decomposition process, the body undergoes some predictable sequence of changes, including temporary muscle stiffness, changes in color, expansion of free gas, generation of scavenging fluid, slippage of the epidermis, destruction of soft tissue, and eventual destruction of the bone^4–6^. (2) Temperature Changes. Currently the most promising estimates are based on recording multiple rectal temperatures or measuring temperatures from the eyes or ears^7–9^. (3) Entomology. Forensic entomology is a discipline that applies entomological and other related arthropod evidence to solve relevant criminal and civil problems in judicial practice. The insects used to research are those reside on a corpse especially. PMI inference is one of the most primary and important applications of forensic entomology. PMI was mainly inferred from insect ontogeny, insect community succession and insect metabolites and gene expression^10–12^. The new PMI inference methods mainly include postmortem chemistry, molecular methods, microbial assay and so on. The integrity and content of DNA, RNA and protein decreases in a time-dependent manner. However, these reductions differ between tissue and environmental conditions, such as temperature and humidity^13–15^. The emergence of microgenomics has significantly improved microbial detection. Therefore, the value of microbes in forensic expertise has attracted more attention. For example, Metcalf *et al*.^16^ established a microbial clock providing an accurate estimate of PMI in a mouse model system. Johnson *et al*.^17^ have successfully demonstrated that skin microbiota is a promising tool in forensic death investigations. In addition, Debruyn *et al*.^18^ documented postmortem changes in human gut bacterial communities. Adserias *et al*.^19^ monitored the oral microbiota of decaying bodies daily to identify characteristic bacterial taxa. Altogether the traditional methods are negatively disturbed by the external environment or the subjective judgment of the examiners. Because of the fixed mathematical models, the microbial method is less affected by the subjective judgment of the inspector and is relatively more reliable. Thus, the method of estimating PMI by microorganism can now serve as an auxiliary tool. Applying changes in microbial communities to infer the PMIs has gradually become a hot topic in forensic research.

Oral cavity is one of the key research fields of human microbial community engineering. It is one of the most abundant areas of human microbial community and the second largest human complex after colon^20^. About 1000 bacterial species have been found in the oral microbial community, with representatives from the phyla Actinomycetes, Proteobacteria, Firmicutes, Bacteroidetes, Synergists, Spirochetes and Aponeurophytes^21^. What’s more, oral and gastrointestinal tract autochthonous microbiome plays a key role in decomposition^22^. Thus, the method of estimating PMI by microorganism can now serve as an auxiliary tool. However, few studies have focused on the ecological changes of oral microbiome which has the potential to be one of the inferred PMI indicators.

This study aimed to investigate the correlation of oral microbes with PMI in mouse models. It is expected to find species which are significantly correlated with PMI at different biological classification levels and to establish a simple linear regression model that can be helpful to estimate PMI more accurately.

## Results

In total, we collected 24 oral cavity samples. Only one male mouse sample with a PMI of 0 hr was not successfully sequenced, and the remaining 23 samples were successfully sequenced. After sequence quality filtering, removal of failed samples and low numbers of sequences, using the IonS5^TM^ XL sequence dataset which includes the 1722677 16sRNA sequences, 1455 OTUs were obtained. 16s rRNA sequencing data has been submitted to the SRA database and the accession number is SRP194019.

### Bacterial community change at phylum level during decomposition

**Figure 1:**
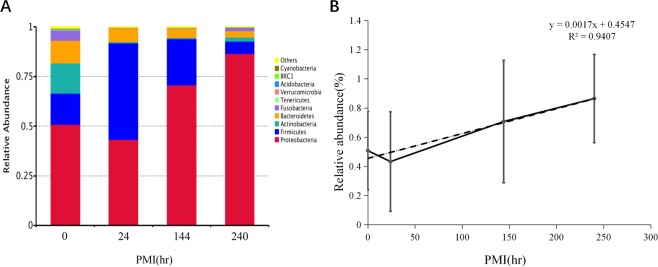
(**A**) Relative abundance of phyla of bacteria with different PMI. (**B**) Relative abundance changes of Proteobacteria had high positive linear relationship with PMI.

The top ten bacteria with the highest abundance in each group at different taxonomic level were analyzed. At the phylum level, within 240 hrs after the death of mice, the Proteobacteria and Firmicutes always occupied the dominant position. Proteobacteria displayed a tendency of decreasing first and then increasing, while phylum of Firmicutes showed a tendency of first increasing and then decreasing. Actinobacteria and Bacteroidetes are the third and fourth highest phyla, which decreased with the increasing of PMI (Fig. 1A). It was found that relative abundance changes of Proteobacteria showed a positive linear correlation with PMI, (PCCs)Pearson correlation coefficient = 0.970, p = 0.030^*^ (Fig. 1B).

**Figure 2:**
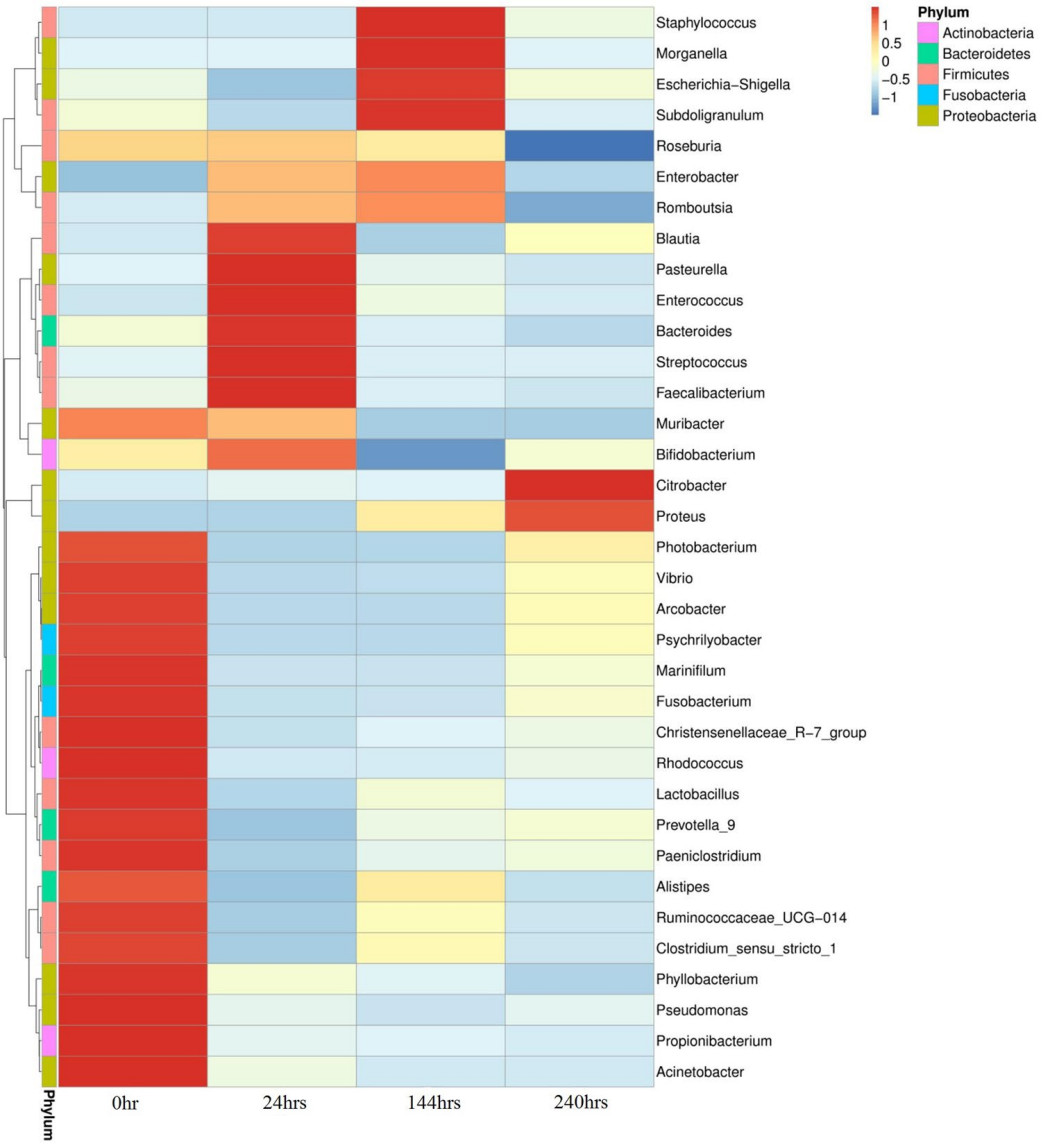
Heat map of top 35 genera of relative abundance of mice oral microbes. The relative abundance of oral microbes in mice varies with PMI.

It was worth noting that at different postmortem intervals, the oral microbes of mice are basically dominated by the same phyla, but the genus of microbes are different (Fig. 2). When the postmortem interval was 0 hr, it was Proteobacteria (*Acinetobacter*, *Pseudomonas*, *Phyllobacterium*, *Photobacterium*, *Vibrio*, *Arcobacter*, *Muribacter*), Actinobacteria (*Propionibacterium*, *Rhodococcus*), Firmicutes (*Ruminococcaceae_UCG-014*, *Clostridiumsensu_stricto_1*, *Paeniclostridium*, *Lactobacillus*, *Christensenelaceae_R-7_Group*), Bacteroidetes (*Alistipes*, *Prevotella _9*, *Marinitilum*) and Fusobacteria (*Fusobacterium*, *Psychrilyobacter*) that basically constituted the mice oral microbes. On the PMI of 24 hrs, mice oral microbes mainly included Firmicutes (*Blautia*, *Enterococcus*, *Streptococcus*, *Faecalbacterium*), Proteobacteria (*Pasteurella*), Bacteroidetes (*Bacteroides*), Actinobacteria (*Bifidobacterium*). When the PMI is 144 hrs, mice oral microbes was principally made up of Actinobacteria (*Staphylococcus*, Subdoligranulum, *Romboutsia*) and Proteobacteria (*Morganella*, *Escherichia shigella*, *Enterobacter*). In addition, it was Proteobacteria (*Citrobacter*, *Proteus*) which chiefly comprised oral microbes on the PMI of 240 hrs.

### Bacterial community at class-level change during PMI decomposition

**Figure 3:**
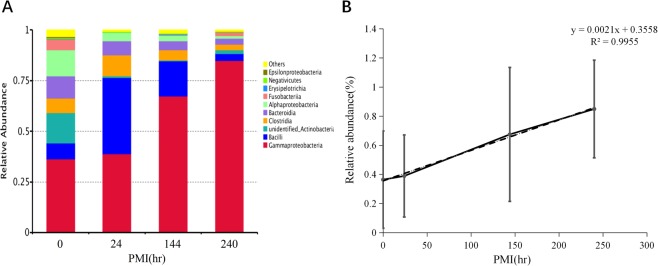
(**A**) Relative abundance of classes of bacteria with different PMI. (**B**) Relative abundance changes of Gamma-proteobacteria had positive strong linear relationship with PMI.

At the class level, when the PMI was 0 hr, the distribution of oral microbes was relatively uniform, but with the increase of PMI, the relative abundance of Gamma-proteobacteria was increased, moreover, Alpha-proteobacteria and Bacteroidia has decreased. Bacilli and Clostridia showed a trend of first increasing and then decreasing with the change of PMI (Fig. 3A). Relative abundance of Gamma-proteobacteria showed a strong positive linear correlation with PMI (PCCs = 0.998, ^**^p = 0.002) (Fig. 3B).

### Bacterial community at order-level change during PMI decomposition

**Figure 4:**
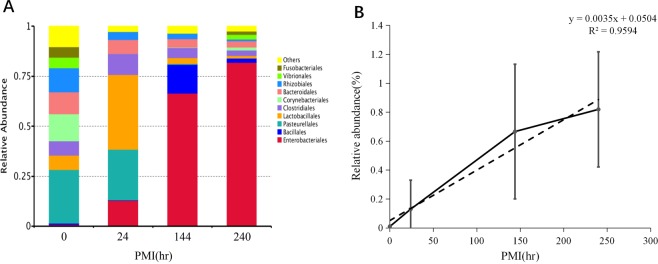
(**A**) Relative abundance of orders of bacteria with different PMI. (**B**) Relative abundance changes of Enterobacteriales had high positive linear relationship with PMI.

When the biological level was order, the relative abundance of Entero-bacteriales increased with the rise of PMI, while the relative abundance of Pasteurellales, Bacteroidales and Rhizobiales decreased with the increase of PMI, and the relative abundance of Lactobacillales increased first and then decreased with the increase of PMI (Fig. 4A). The positive linear relationship between the relative abundance of Enterobacteriales and PMI is the strongest at the level of order (PCCs = 0.979, ^*^p = 0.021) (Fig. 4B).

### Bacterial community changes at the family level during PMI decomposition

**Figure 5:**
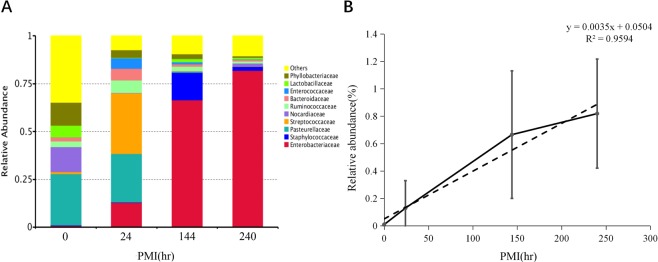
(**A**) Relative abundance of families of bacteria with different PMI. (**B**) Relative abundance changes of Enterobacteriaceae had high positive linear relationship with PMI.

At the family level, the relative abundance of Enterobacteriaceae increased with the rise of PMI though the relative abundance of Pasteurellaceaeae and Phyllobacteriaceae decreased. Meanwhile, the relative abundance of Streptococcaceae, Ruminococcaceae and Bacteroidaceae presented a tendency of increasing first and then decreasing (Fig. 5A). We also discovered that the relative abundance of Enterobacteriaceae showed a high positive relationship with PMI (PCCs = 0.979, ^*^p = 0.021) (Fig. 5B).

### Bacterial community changes at the genus level during PMI decomposition

**Figure 6:**
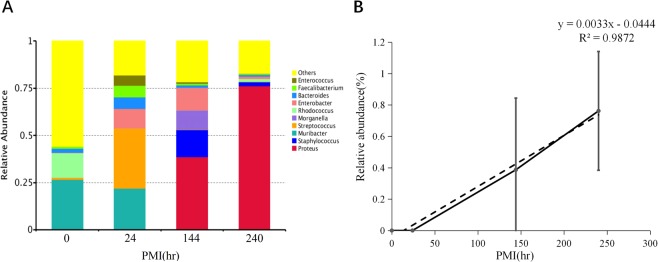
(**A**) Relative abundance of genera of bacteria with different PMI. (**B**) Relative abundance changes of Proteus had strong positive linear relationship with PMI.

At the biological level of genus, with the rise of PMI, the relative abundance of *Proteus* increased, while the relative abundance of *Muribacter* and *Phyllobacterium* increased first and then decreased (Fig. 6A). Relative abundance of *Proteus* showed strong positive linear relationship with PMI (PCCs = 0.994, ^**^p = 0.006) (Fig. 6B).

### Diversity analysis of bacterial community succession

**Figure 7:**
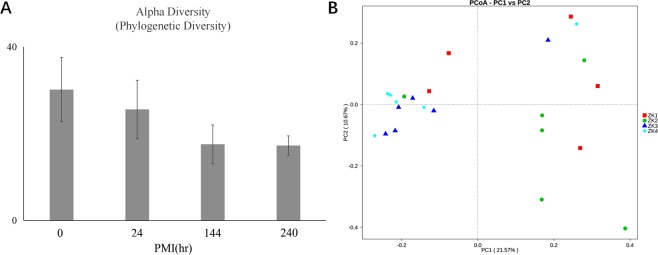
Diversity analysis of oral microbial communities of different PMI. (**A**) Phylogenetic distance (PD) alpha diversity for microbial communities at each group. (**B**) PCoA plot based on unweighted UniFrac distances displaying microbial communities change during death.

The alpha diversity of oral microbial communities in mice decreased slightly with the increase of PMI (Fig. 7A). The communities composition of oral microbes on the PMI of 144 hrs was superficially similar to that when the PMI was 240 hrs (Fig. 7B). It indicated that with the increase of PMI, the individual difference decreased gradually and the similarity of microbial communities between different PMI raised. Meanwhile, there were significant differences in microbial communities composition between the two groups besides 240 hrs vs 144 hrs and 24 hrs vs 0 hr. When the death time was relatively far apart, there was a significant difference in communities composition (Table 1).

**Table 1:** Adonis Group Difference Analysis of microbial communities’ composition between different PMI.

Vs_ group	F. Model	R^2^	Pr (>F)
240 hrs-24 hrs	7.3247	0.42279 (0.57721)	0.005
240 hrs-0 hr	5.7658	0.39048 (0.60952)	0.004
240 hrs-144 hrs	1.3718	0.12063 (0.87937)	0.265
24 hrs-0 hr	2.1598	0.19354 (0.80646)	0.065
24 hrs-144 hrs	2.9687	0.22891 (0.77109)	0.009
0 hr-144 hrs	2.4809	0.21609 (0.78391)	0.012

F. Model is F test value. R^2^ indicates the interpretation degree of sample difference in different groups, that is, the larger the ratio of group variance to total variance is, the higher the interpretation degree of the group to the difference is. In addition, the Pr is P value, and less than 0.05 means that the reliability of this test is high. The value of the residuals in parentheses.

In conclusion, at each taxonomic level, a bacterium with significant correlation with PMI changes was respectively found, and linear regression models between relative abundance and PMI were constructed (Table 2). The larger the R^2^, the better the fitting degree of the linear regression equation. Therefore, Gamma-proteobacteria and *Proteus* were the best candidates that can be used to infer PMI, especially late PMI.

**Table 2:** The classification and linear regression equation of species which have significant correlation between relative abundance with PMI change.

Species	Family	Order	Class	Phylum	Linear regression equation	R^2^
Proteobacteria	—	—	—	—	y = 0.0017x + 0.4547	0.94
Gammaproteobacteria	—	—	—	Proteobacteria	y = 0.0021x + 0.3558	0.99
Enterobacteriales	—	—	Gammaproteobacteria	Proteobacteria	y = 0.0035x + 0.0504	0.96
Enterobacteriaceae	—	Enterobacteriales	Gammaproteobacteria	Proteobacteria	y = 0.0035x + 0.0504	0.96
Proteus	Enterobacteriaceae	Enterobacteriales	Gammaproteobacteria	Proteobacteria	y = 0.0033x − 0.0444	0.99

The y represents PMI (hr) and x represents the relative abundance (%) of species.

## Discussion

In this study, we used the mouse model, one of the most important model organisms, to develop a potential tool for estimating the PMI with the succession of oral microbial community. Large number of samples allowed us to perform replicate experiments with a large number of samples to minimize experimental error and assess to what extent the intra-individual variation of microbiota that we know is present in living humans and other mammals^23^.

The oral cavity was chosen as the sampling point for microbes. Compared with intestinal sampling which needs dissection, oral sampling is much more convenient. The Human Microbiome Project observes that human stool harbors a rich microbiome, while the oral cavity is relatively limited^24^. Furthermore, Guo *et al*. observed the same phenomenon in samples collected from live rats^25^. Therefore, the analysis of oral microbes simplifies our analytical work and helps to find the target species that are associated with PMI. Meanwhile, oral microbes are an important part of the human microbiota, few articles focus on the relationship between oral microbial community succession and PMI. In our study, the dominant phyla in the oral cavity of the mice were Proteobacteria, Firmicutes, Actinobacteria and Bacteroidetes when PMI were 0 hr, similar to those in the mouth of healthy humans, as reported by the HMP^24^. Since the oral cavity is in direct contact with the outside environment, the change of microbiome is easy to be influenced by the external factors such as temperature and air humidity, we tried to keep the same experimental conditions by controlling the temperature and humidity and other controllable factors in order to minimize the impact of the external environment. In this case, the changes of oral microbes were analyzed in order to obtain basic and relatively standardized results. Further investigation regarding the external factors needs to be done in the future.

From the current data, we have obtained one bacterium at each taxonomic level that is associated with the change in PMI, namely, Proteobacteria, Gamma-proteobacteria, Entero-bacteriales, Enterobacteriaceae and *Proteus*. Interestingly, these candidates all belong to the Proteobacteria. Proteobacteria is the largest phylum in the bacterial kingdom. They are all Gram-negative bacteria, which have both aerobic and anaerobic bacteria. Proteobacteria are usually associated with the decay of meat and have been found on the hides of slaughtered animals^26^. Besides, oral microbiome mainly includes aerobic bacteria and facultative anaerobic bacteria. *Proteus*, a dominant bacterium, is also facultative anaerobic, which may be related to the closed state of oral cavity when mice die. Therefore, the effect of oral closed state on oral microbial changes when mice die cannot be ignored. For another, the relative abundance of these candidates increases with the increase of PMI, but the R^2^ of microbes with PMI between adjacent classification levels are not the same, except the order and family level. It may be due to the presence of other influential species in addition to the dominant species. It should be noted that Enterobacteriales contains only one family of Enterobacteriaceae, so the linear relationship between relative abundance of them with PMI is the same.

Unlike previous reports that the relative abundance of Firmicutes decreased first and then increased with PMI in oral cavity of rats and human^19,25^, our data demonstrated that it increased first and then decreased with PMI. The relative abundance of Firmicutes increased at 24 hrs of PMI and then decreased, similar to Bacilli, Lactobacillales, Streptococcaceae and *Streptococcus*. 24 hrs is the time point to distinguish early PMI from late PMI. As a conclusion, the community composition of oral microbes changed a lot before and after 24 hrs. Meanwhile, all the bacteria with turning change, which means that the change of relative abundance of species with prolongation of PMI is first decreasing then increasing or first increasing and then decreasing, belong to Firmicutes. Although Firmicutes was reported as one of the main phyla in the human gut (intestine, rectum and cecum) and fecal samples^24,27–30^, unfortunately, it does not have a significant linear relationship with PMI. In our results, the relative abundance of Actinobacteria has been decreased with the increase of PMI, which is consistent with the previous reports in oral cavity of rats and human^19,25^. Actinobacteria are widely distributed in terrestrial and aquatic ecosystems, especially in soil, which play an important role in the recycling of refractory biomaterials through decomposition and humus formation^31^. We also found that Gamma-proteobacteria were predominant throughout the postmortem interval in mice, which was concordant with that in preceding report^25^. The Gamma-proteobacteria exhibit different metabolic abilities and are involved in the decomposition of more complex molecules^32^. Taken together, Gamma-proteobacteria may be an important contributor to the decomposition process. Enterobacteriaceae, an important component of intestinal microbes, was found to be dominant in the oral microbial community in the late PMI. There was also a significant linear relationship between its relative abundance and PMI. Metcalf *et al*.^16^ also found that Enterobacteriaceae bacteria such as Serratia, Escherichia, Klebsiella and Proteus became abundant after rupture. They were widely regarded as opportunistic pathogens related to sewage and animal substances. Whether the combination of the Enterobacteriaceae in oral cavity and abdominal cavity can improve the inferential accuracy of PMI is worth studying in the future. The relative abundance of *Proteus* was very low in the early PMI and higher in the microbiome of the late PMI, which indicated that *Proteus* was more suitable for the prediction of the late PMI. The same conclusion was reached in rats and swines^25,33^. For another, Metcalf *et al*.^34^ found that the mice decomposed in three different soil types, and the error rate was as low as +/2–3 days within 25 days after death. Moreover, Ismail *et al*.^35^ demonstrated that under the same conditions, the dead microbiome of different organs in the same corpse is very similar. Therefore, the accuracy of our results should be similar to the previous reports, but the accurate accuracy needs to be further verified by furture experiments.

In short, Gamma-proteobacteria and *Proteus* are the best potential candidates of oral microbial communities for PMI estimation. Since Enterobacteriaceae is also present in the intestine, it can be considered in combination with its changes in the intestine to infer PMI.

## Materials and Methods

### Experimental set-up and sample collection

All animal experimental procedures and protocols were conducted in accordance with Chinese legislation on the Use and Care of laboratory animals, and were approved by the Animal Ethics Committee (Institutional Animal Care and Use Committee of the East China University of Science and Technology). A total of 24 adult mice (strain ICR) were purchased from Shanghai Slac Laboratory Animal Company (Shanghai, China). The laboratory experiment was performed at East China University of Science and Technology in Shanghai, China. Mice were humanely sacrificed using CO_2_ gas followed by cervical dislocation and placed in clean cartons with sterile cotton gauze and UV-sterilized padding which were used for the absorption of the spoilage liquids produced by corpses in the experiments. Air temperature during the decomposition changed little with average temperature of 22.4 °C. Relative humidity ranged from 33% to 41%, (average 37%).

We separately sampled three female mice and three male mice across four points including 0 hr, 24 hrs, 144 hrs and 240 hrs. According to the previous studies^16,25,34^,24 h is the cut-off point between the early PMI and the late PMI, and many changes in the death process occur after 24 h. Then we divided the remaining nine days into four and five days. So the PMI we choosed was 0 hr, 24 hrs, 144 hrs and 240 hrs. High temperature sterilized cotton swabs wetted with aseptic water were used to wipe the oral cavity and transferred to sterile Eppendorf tube in duplicate. Four sets of samples were named as 0 hr, 24 hrs, 144 hrs and 240 hrs.

### DNA extraction, PCR amplification, and next-generation sequencing

DNA was extracted from the collected swab samples using the classical CTAB method^36^. An agarose gel electrophoresis was used to detect the purity and concentration of DNA. Samples with appropriate amount were taken into the Eppendorf tubes, and the samples were diluted with aseptic water to 1 ng/μL.

For sequencing, total genomic DNA was subjected to PCR application targeting an informative portion of the 16s rRNA variable region 3(V3) and variable region 4(V4) using the bacterial primer set 341F(CCTAYGGGRBGCASCAG)/806R(GGACTACNNGGGTATCTAAT).

The sequencing operation was completed by Novegene Co., LTD (Beijing, China). The sequencing platform was IonS5^TM^ XL. The single-end method was used to construct a small fragment library for single-terminal sequencing.

### 16s rRNA sequencing data processing

Raw reads were obtained by discarding low quality part and cutting barcodes and primer sequence using Catadapt (Version 1.9.1)^37^. Chimeric sequences were detected for alignment with Gold database by UCHIME Algorithm and removed in order to get clean reads^38,39^.

### OTU clustering and species annotation

Operational taxonomic units (OTUs) defined by a 97% identity were picked using the Uparse (Version 7.0.1001)^40^. Meanwhile, the representative sequence, chosen as the most abundant in each OTUs, were submitted to the Mothur in order to acquire the assignment and the abundance of each OUT using the SILVA’s SSUrRNA database at different taxonomic levels: phylum, order, family, genus and species (Threshold value was 0.8–1)^41,42^. Finally, the data of each sample are homogenized based on the minimum amount of data in the sample. The subsequent Alpha diversity analysis and Beta diversity analysis are based on the data after homogenization.

### 16s rRNA data analysis

According to the result of species annotation, the top ten species with the highest abundance in each group at different taxonomic level were selected and made into the columnar accumulative graph so that the samples could be viewed directly in order that we can visually view the relative abundance of the species and their proportions at different classification levels. We analyzed whether there were species at different taxonomic levels that had a direct linear relationship with PMI and plotted a linear regression plot. Moreover, the top 35 genera of abundance are selected for clustering and plotted as heat maps.

The shannon diversity and phylogenetic diversity measures were used to estimate alpha diversity. The beta diversity model was performed by performing a principal coordinate analysis (PCOA) based on unweighted single fraction distance. These analyses were performed using the QIIME software (Version 1.9.1) and R software (Version 2.15.3). Additionally, we tested for significant changes in microbial communities between different PMI with Adonis analysis^43,44^.

## References

[1] Brooks, J. W. & Sutton, L. In Veterinary Forensic Pathology, Volume 1 43–63 (Springer, 2018).

[2] Saks MJ, Koehler JJ (2005). The coming paradigm shift in forensic identification science. Science.

[3] Brooks JW (2016). Postmortem Changes in Animal Carcasses and Estimation of the Postmortem Interval. Veterinary Pathology.

[4] Saukko, P. & Knight, B. Knight’s Forensic Pathology. 3rd ed. New York: Oxford University Press, 2004.

[5] Perper, J. Time of death and changes after death, Part 1. *Medicolegal Investigation of Death* (2006).

[6] Dolinak, D., Matshes, E. & Lew, E. Forensic Pathology (2005).

[7] Rodrigo MR (2016). A Nonlinear Least Squares Approach to Time of Death Estimation Via Body Cooling. Journal of Forensic Sciences.

[8] Baccino E (1996). Outer ear temperature and time of death. Forensic Science International.

[9] Eric B, Cristina C, Christine J, Joel P, Laurent M (2007). Cooling rates of the ear and brain in pig heads submerged in water: implications for postmortem interval estimation of cadavers found in still water. Am J Forensic Med Pathol.

[10] Castro CPE, García MD, Silva PMD, Silva IFE, Serrano A (2013). Coleoptera of forensic interest: A study of seasonal community composition and succession in Lisbon, Portugal. Medical & Veterinary Entomology.

[11] Villet MH (2010). Forensic Entomology: The Utility of Arthropods in Legal Investigations. African Entomology.

[12] Tarone AM, Foran DR (2011). Gene expression during blow fly development: improving the precision of age estimates in forensic entomology. Journal of Forensic Sciences.

[13] Sampaio-Silva F, Magalhães T, Carvalho F, Dinis-Oliveira RJ, Silvestre R (2013). Profiling of RNA degradation for estimation of post morterm interval. PloS one.

[14] Itani M, Yamamoto Y, Doi Y, Miyaishi S (2011). Quantitative analysis of DNA degradation in the dead body. Acta Med Okayama.

[15] Poloz YO, O’Day DH (2009). Determining time of death: temperature-dependent postmortem changes in calcineurin A, MARCKS, CaMKII, and protein phosphatase 2A in mouse. International journal of legal medicine.

[16] Metcalf JL (2013). A microbial clock provides an accurate estimate of the postmortem interval in a mouse model system. elife.

[17] Johnson HR (2016). A machine learning approach for using the postmortem skin microbiome to estimate the postmortem interval. PloS one.

[18] DeBruyn JM, Hauther KA (2017). Postmortem succession of gut microbial communities in deceased human subjects. PeerJ.

[19] Adserias‐Garriga J (2017). Dynamics of the oral microbiota as a tool to estimate time since death. Molecular oral microbiology.

[20] Wade WG (2013). The oral microbiome in health and disease. Pharmacological research.

[21] Dewhirst FE (2010). The human oral microbiome. Journal of bacteriology.

[22] Janaway, R. C., Percival, S. L. & Wilson, A. S. Decomposition of Human Remains (2009).

[23] Costello EK (2009). Bacterial community variation in human body habitats across space and time. Science.

[24] Huttenhower C (2012). Structure, function and diversity of the healthy human microbiome. nature.

[25] Guo J (2016). Potential use of bacterial community succession for estimating post-mortem interval as revealed by high-throughput sequencing. Scientific reports.

[26] Gill C, Newton K (1978). The ecology of bacterial spoilage of fresh meat at chill temperatures. Meat science.

[27] Eckburg PB (2005). Diversity of the human intestinal microbial flora. science.

[28] Bäckhed F, Ley RE, Sonnenburg JL, Peterson DA, Gordon JI (2005). Host-bacterial mutualism in the human intestine. science.

[29] Turnbaugh PJ (2006). An obesity-associated gut microbiome with increased capacity for energy harvest. nature.

[30] Li M (2008). Symbiotic gut microbes modulate human metabolic phenotypes. Proceedings of the National Academy of Sciences.

[31] Tuomisto S, Karhunen PJ, Pessi T (2013). Time-dependent post mortem changes in the composition of intestinal bacteria using real-time quantitative PCR. Gut pathogens.

[32] Dickson GC, Poulter RT, Maas EW, Probert PK, Kieser JA (2011). Marine bacterial succession as a potential indicator of postmortem submersion interval. Forensic science international.

[33] Pechal JL (2014). The potential use of bacterial community succession in forensics as described by high throughput metagenomic sequencing. International Journal of Legal Medicine.

[34] Metcalf JL (2016). Microbial community assembly and metabolic function during mammalian corpse decomposition. Science.

[35] Can I, Javan GT, Pozhitkov AE, Noble PA (2014). Distinctive thanatomicrobiome signatures found in the blood and internal organs of humans. Journal of microbiological methods.

[36] Porebski S, Bailey LG, Baum BR (1997). Modification of a CTAB DNA extraction protocol for plants containing high polysaccharide and polyphenol components. Plant molecular biology reporter.

[37] Martin M (2011). Cutadapt removes adapter sequences from high-throughput sequencing reads. EMBnet. journal.

[38] Edgar RC, Haas BJ, Clemente JC, Quince C, Knight R (2011). UCHIME improves sensitivity and speed of chimera detection. Bioinformatics.

[39] Haas BJ (2011). Chimeric 16S rRNA sequence formation and detection in Sanger and 454-pyrosequenced PCR amplicons. Genome research.

[40] Edgar RC (2013). UPARSE: highly accurate OTU sequences from microbial amplicon reads. Nature methods.

[41] Wang Q, Garrity GM, Tiedje JM, Cole JR (2007). Naive Bayesian classifier for rapid assignment of rRNA sequences into the new bacterial taxonomy. Appl. Environ. Microbiol..

[42] Quast C (2012). The SILVA ribosomal RNA gene database project: improved data processing and web-based tools. Nucleic acids research.

[43] Anderson MJ (2001). A new method for non‐parametric multivariate analysis of variance. Austral ecology.

[44] McArdle BH, Anderson MJ (2001). Fitting multivariate models to community data: a comment on distance‐based redundancy analysis. Ecology.

